# Come together over me: Cells that form the dermatocranium and chondrocranium in mice

**DOI:** 10.1002/ar.25295

**Published:** 2023-07-27

**Authors:** M. Kathleen Pitirri, Joan T. Richtsmeier, Mizuho Kawasaki, Abigail P. Coupe, Susan Motch Perrine, Kazuhiko Kawasaki

**Affiliations:** ^1^ Department of Anthropology The Pennsylvania State University University Park Pennsylvania USA

**Keywords:** cranial neural crest, OSX, osteoblast differentiation, paraxial mesoderm, RUNX2, SOX9, skeletogenesis

## Abstract

Most bone develops either by intramembranous ossification where bone forms within a soft connective tissue, or by endochondral ossification by way of a cartilage anlagen or model. Bones of the skull can form endochondrally or intramembranously or represent a combination of the two types of ossification. Contrary to the classical definition of intramembranous ossification, we have previously described a tight temporo‐spatial relationship between cranial cartilages and dermal bone formation and proposed a mechanistic relationship between chondrocranial cartilage and dermal bone. Here, we further investigate this relationship through an analysis of how cells organize to form cranial cartilages and dermal bone. Using Wnt1‐Cre2 and Mesp1‐Cre transgenic mice, we determine the derivation of cells that comprise cranial cartilages from either cranial neural crest (CNC) or paraxial mesoderm (PM). We confirm a previously determined CNC‐PM boundary that runs through the hypophyseal fenestra in the cartilaginous braincase floor and identify four additional CNC‐PM boundaries in the chondrocranial lateral wall, including a boundary that runs along the basal and apical ends of the hypochiasmatic cartilage. Based on the knowledge that as osteoblasts differentiate from CNC‐ and PM‐derived mesenchyme, the differentiating cells express the transcription factor genes *RUNX2* and osterix (*OSX*), we created a new transgenic mouse line called R2Tom. R2Tom mice carry a tdTomato reporter gene joined with an evolutionarily well‐conserved enhancer sequence of *RUNX2*. R2Tom mice crossed with Osx‐GFP mice yield R2Tom;Osx‐GFP double transgenic mice in which various stages of osteoblasts and their precursors are detected with different fluorescent reporters. We use the R2Tom;Osx‐GFP mice, new data on the cell derivation of cranial cartilages, histology, immunohistochemistry, and detailed morphological observations combined with data from other investigators to summarize the differentiation of cranial mesenchyme as it forms condensations that become chondrocranial cartilages and associated dermal bones of the lateral cranial wall. These data advance our previous findings of a tendency of cranial cartilage and dermal bone development to vary jointly in a coordinated manner, promoting a role for cranial cartilages in intramembranous bone formation.

## 1 INTRODUCTION

Prior to the development of cranial bones, sutures, or synchondroses, a skull composed entirely of cartilage called the cranial endoskeleton forms to support the brain and other cranial sense organs. The cranial endoskeleton includes the cartilaginous chondrocranium and pharyngeal skeleton that form prior to the cranial dermal skeleton (dermatocranium) (Kawasaki & Richtsmeier, 2017). The cranial endoskeleton and dermatocranium evolved as distinct systems and form separately during embryogenesis but merge over developmental time such that most modern vertebrate skulls are composite structures formed by the union of the cranial endo and dermal skeletons. Though well studied by evolutionary biologists and comparative zoologists as a functioning skeletal organ (de Beer, 1937; Goodrich, 1930), the chondrocranium has garnered relatively less attention in the field of developmental biology. In mammals, much of the chondrocranium is transient, undergoing endochondral ossification or disappearing as dermal bones form (Moore, 1981), so its role in skull morphogenesis is not well understood. However, the association of these two skeletal systems has been maintained for over 470 million years of evolutionary history (Janvier, 1993, 2015), except in chondrichthyes that secondarily lost their dermal skeleton (Schultze, 1993), suggesting that the chondrocranium is essential to cranial development in vertebrates (Kawasaki & Richtsmeier, 2017).

The developing bony skull is composed of elements of the dermatocranium that mineralize intramembranously and of the endoskeleton that mineralize perichondrally and/or endochondrally (Moore, 1981). Endochondral bone has a close and well‐studied developmental relationship with associated cartilaginous anlagen. But dermal bone that mineralizes intramembranously and chondrocranial cartilage are established separately during embryogenesis and remain separate (Hirasawa & Kuratani, 2015). In brief, elements of the mouse chondrocranium form individually in sequence beginning at embryonic day 12.5 (E12.5), fuse to provide a cartilaginous protective covering for the brain and other sense organs by E15.5, and certain elements begin to dissolve by E16.5 as dermal bone begins to mineralize (Pitirri et al., 2020). Based on our observations of the temporospatial relationship between the disappearance of specific chondrocranial cartilages and the formation of dermal bones (Kawasaki & Richtsmeier, 2017; Pitirri et al., 2020) and the statistical tendency of cranial cartilage and dermal bone development to vary jointly in a coordinated manner (Motch Perrine et al., 2022), we propose that the two systems are linked. To further explore this hypothesis, we provide some background on chondrocranium and skull development and present new data pertaining to the development of cartilages of the cranial vault and their relationship to developing bones of the vault.

### 1.1 Cranial skeletal tissues

Like elements of the appendicular skeleton, cranial skeletal elements develop in a distinct and predictable location in the head and neck, form specific articulations with other elements, serve as attachment points for muscles and tendons, and grow to reach a specific size and shape according to directives accumulated over evolutionary time and the supervision of specific genes in any individual. These patterns of bone and cartilage development are preceded by intricately choreographed processes of cell migration and condensation by mesenchymal cells that originate from varying germ‐ and tissue‐layer sources.

#### 1.1.1 Cell derivation

**FIGURE 1:**
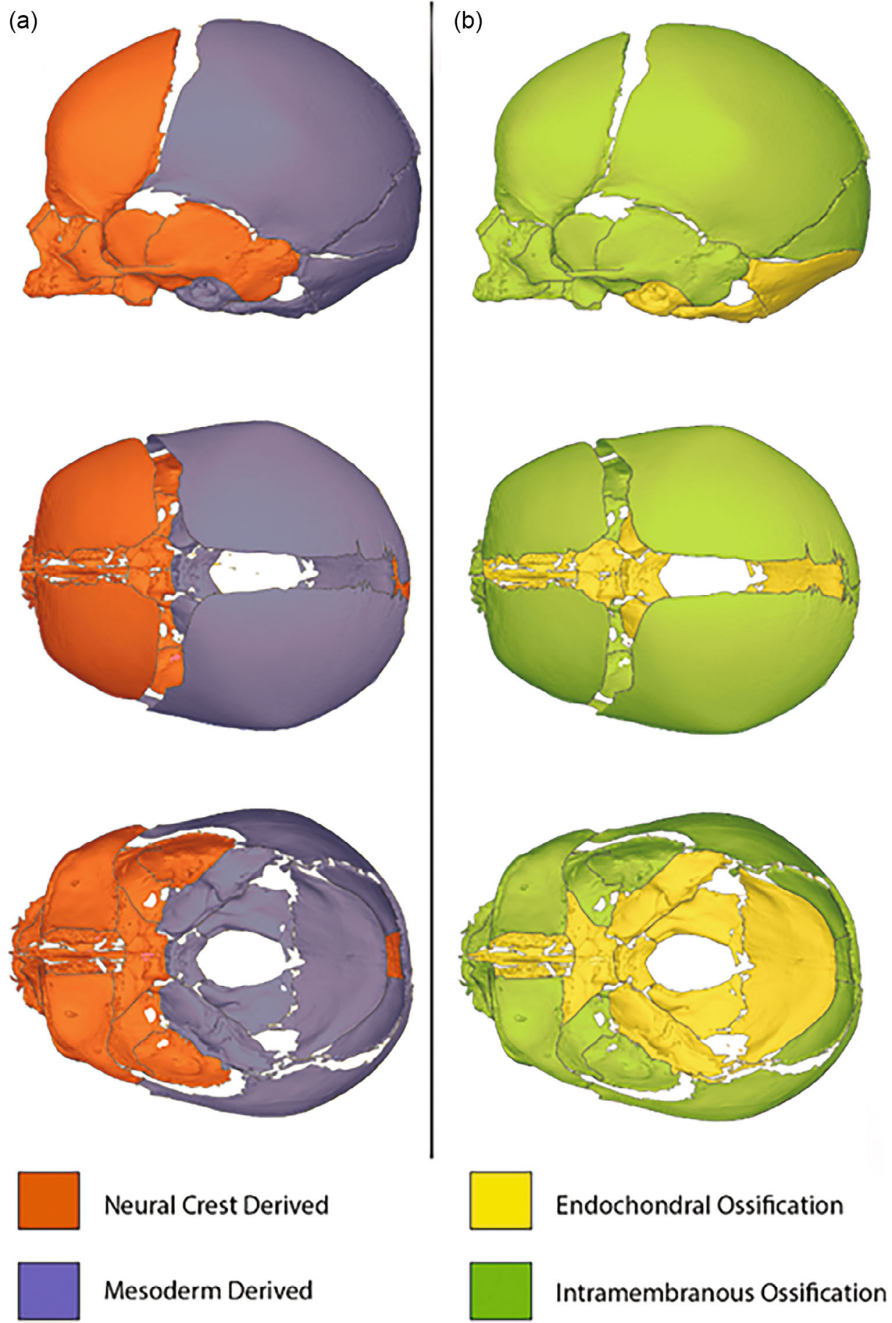
Three‐dimensional (3D) reconstruction of computed tomography (CT) images of a human neonatal cranium. Left lateral view at top, superior view at middle, superior view with bones of the cranial vault removed to show endocranial surface at bottom. Face to the left, occiput to the right in all views. Crania are labeled according to (a) cellular origin of cranial bones: cranial neural crest in orange and mesoderm in blue, and (b) ossification type: intramembranous ossification in green and endochondral ossification in yellow. Adapted from (Flaherty et al., 2016). Original figure drawn by Dr. Kevin Flaherty.

Nearly all postcranial cartilage and bone and some cranial cartilage and bone are derived from the mesoderm germ layer. Bones of the skull derive either from paraxial mesoderm (PM) cells or cells derived from the cranial neural crest (CNC). Both CNC and PM cell populations migrate extensively to diverse locations in the embryo and contribute to an array of different tissue types (Dash & Trainor, 2020; Saykali et al., 2019). Skeletally, CNC cells contribute to bones of the cranial vault, facial skeleton, pharyngeal skeleton, and small regions of the scapula and clavicle (Frisdal & Trainor, 2014; Galea et al., 2021; Huang et al., 1997; Jiang et al., 2002). The distinct PM or CNC origins of the components of the human skull have been inferred primarily from work in chick and mouse (Jiang et al., 2002; Le Douarin & Kalcheim, 1999; McBratney‐Owen et al., 2008) (Figure 1). Available information suggests that the positional origin of neural crest cells from the diencephalon, midbrain, and hindbrain epithelium contributes to their coherence after differentiation (Kontges & Lumsden, 1996; Santagati & Rijli, 2003) and that PM and CNC cell dynamics are coordinated during head morphogenesis (McKinney et al., 2020).

During cranial formation, cells that have migrated and interacted with overlying epithelial cells form emergent chondrogenic and osteogenic condensations. Individual cranial cartilages and bones emerge from cell condensations that originate from changes in the mitotic activity of cells, aggregation of cells towards a center, or failure of cells to move away from a center (Hall & Miyake, 2000). Condensations facilitate differentiation into chondrocytes or osteoblasts that form cranial cartilage or cranial dermal bone, respectively, and comprise the raw material for morphology (Galea et al., 2021; Hall & Miyake, 1995, 2000).

#### 1.1.2 Modes of ossification

Bone matrix is secreted by osteoblasts in both endochondral and intramembranous bone formation (de Crombrugghe et al., 2001; Hartmann, 2009; Karsenty et al., 2009; Lefebvre & Bhattaram, 2010). Endochondral bone formation (Figure 2.1) begins with an aggregation of multipotent mesenchymal cells that differentiate into chondrocyte precursors. These cells subsequently secrete the matrix that composes a cartilaginous model in the shape of the future element. This cartilaginous model is eventually replaced by bone.

Much of our knowledge of endochondral ossification comes from the study of long bones of the post‐cranial skeleton, in which the primary ossification center forms around chondrocytes that undergo hypertrophy (Figure 2.1). The initial bone (bone collar) forms on the cartilage surface near hypertrophic chondrocytes (perichondral ossification), which is followed by mineralization of the cartilage matrix around the hypertrophic chondrocytes. Subsequently, the bone collar is resorbed by osteoclasts, which allows invasion of vasculature and osteoblast precursors. The invading vasculature also transports osteoclasts that work to remove the mineralized cartilage matrix while differentiated osteoblasts secrete bone matrix (osteoid) onto resorbed cartilage matrix (Karsenty et al., 2009; Lefebvre & Bhattaram, 2010). Some of these osteoblasts embedded in the matrix differentiate into osteocytes. As the primary ossification center develops, part of the remaining cartilage forms the growth plates at both ends of most long bones (Blumer, 2021). The process of endochondral ossification is similar for cranial bones, though cranial growth plates can be organized differently (Cendekiawan et al., 2010).

**FIGURE 2:**
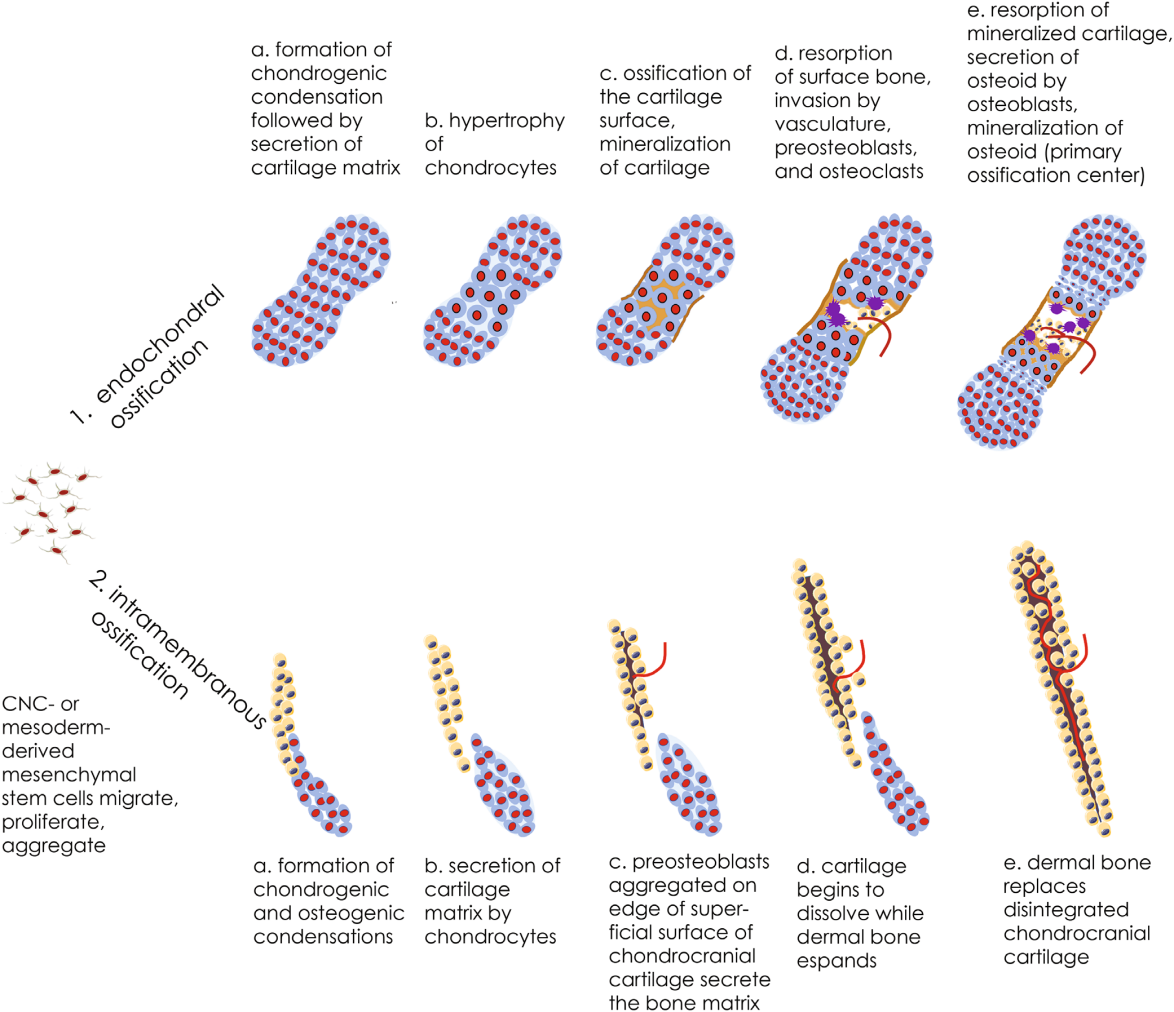
The major steps of cranial endochondral and intramembranous ossification. In endochondral ossification (1a–e), after aggregated prechondrogenic cells differentiate into chondrocytes, a cartilage matrix is produced to form a cartilaginous element in the shape of the future bone (1a). Select chondrocyte hypertrophy (1b). Peripheral cells surrounding the cartilaginous model form a perichondral cellular surface layer that mineralizes (bone collar), while the cartilage matrix around hypertrophic chondrocytes is mineralized (1c). Bone collar is invaded by blood vessels, which allows the invasion of preosteoblasts and osteoclasts (1d). Mineralized cartilage matrix is resorbed by osteoclasts and invaded by a cohort of bone‐forming cells that secrete osteoid (1e). In intramembranous ossification (2a–e), aggregated chondrogenic and osteogenic cells form condensations (2a). Chondrogenic cells differentiate into chondrocytes and cartilage matrix is produced to form chondrocranial cartilaginous elements, while preosteoblasts form condensations on the edges of the superficial surface of cranial cartilages (2b), where they directly and progressively differentiate into osteoblasts and begin to secrete the bone matrix (2c). As the neighboring chondrocranial cartilage dissolves, some of the osteoblasts secrete osteoid and some osteoblasts embedded in the matrix differentiate into osteocytes (2e).

Cranial bones of the exoskeleton that form by intramembranous ossification (dermal bone) begin as aggregations of mesenchymal cells that directly differentiate into osteoblasts (Figure 2.2) (Hartmann, 2009; Komori et al., 1997). Intramembranous ossification involves the progressive differentiation of osteoblast precursors into osteoblasts, a process that is induced by specific transcription factors (de Crombrugghe et al., 2001; Komori, 2019, 2020; Lefebvre & Dvir‐Ginzberg, 2017). Osteoblasts subsequently secrete osteoid, and, as some of these cells become embedded in the mineralized bone matrix, they differentiate into osteocytes (Franz‐Odendaal & Hall, 2006). Though intramembranous ossification is often defined as occurring without a cartilaginous model, our data show that preosteoblasts that contribute to the formation of most cranial dermal bones form condensations adjacent to an edge of their underlying, associated cranial cartilages that are transient and subsequently degraded (Pitirri et al., 2020).

#### 1.1.3 The fate of cartilages that form the chondrocranium

As noted above, chondrocytes and osteoblasts are derived from similar mesenchymal condensations during endochondral ossification (Day et al., 2005; Hill et al., 2005). Condensations for individual cranial bones and cartilages develop at their own pace, establishing an element‐specific time of condensation, location of condensation, and initial shape. Chondrocyte precursors that compose condensations during cartilage formation, including those condensations that serve as the cartilage model of endochondral ossification (de Crombrugghe et al., 2001), begin to differentiate into chondroblasts that secrete the cartilage matrix prior to the differentiation of osteoblasts that secrete the matrix of the overlapping dermal bone or the collar of endochondral bone.

In mice, precartilaginous condensations of the chondrocranium form individual elements beginning with the appearance of the parachordal cartilages around embryonic day 12.5 (E12.5) (Kawasaki & Richtsmeier, 2017). Though the timing of individual chondrification sequences is highly variable, chondrocranial cartilages form rapidly and coalesce, so that by E15.5 the chondrocranium provides an intricate protective covering for the brain and other sense organs. After this, chondrocranial cartilages have diverse fates. Some cranial cartilages remain cartilage in the adult (e.g., nasal septum). Others, like the parachordal cartilage, ossify endochondrally. Finally, the matrix of certain unmineralized cartilages like the ala orbitalis is enzymatically degraded. Though this process is not thoroughly understood, it has been shown that membrane‐type 1 matrix metalloproteinase (MT1‐MMP)–dependent degradation of cranial cartilages, coupled with apoptosis of nonhypertrophic chondrocytes, mediates remodeling of some cranial cartilages into other tissues (Holmbeck et al., 2003) and that some nasal capsule cartilage tissue appears to break apart, while others are at least partially resorbed by chondroclasts (Smith et al., 2021). In the case of chondrocranial cartilages that we have studied, another potential step in this process is our discovery that as cranial cartilages dissolve, mineralization of dermal bone progresses on the external surface of their perimeters.

### 1.2 Problem formulation

As described above, the relationship between cranial cartilages and endochondral bone is well established. Intramembranous bones of the skull ossify directly from pre‐osteogenic condensations and are often defined as forming “without a cartilaginous model” (Gruneberg & Wickramaratne, 1974). We have previously identified a temporospatial relationship between specific chondrocranial elements and dermal bones of the cranial vault and facial skeleton (Kawasaki & Richtsmeier, 2017; Pitirri et al., 2020) and proposed a link between cranial cartilage and cranial dermal bone development based on these observations and statistical associations (Motch Perrine et al., 2022). Using laboratory mice as a valuable system for understanding the relationship between the chondrocranium and dermatocranium, we combine what is known of the chondrocranial cartilages of the lateral wall (the ala orbitalis, tectum transversum, orbitoparietal commissure, and parietal plate) and their associated dermal bones (frontal, parietal, and squamosal) with new data. We show how skeletogenic cell condensations residing in the potential space between the neuroepithelium and surface ectoderm form emergent chondrocranial cartilages and dermal bones of the lateral wall, with chondrocytes differentiating to form cartilages just prior to dermal bone formation. We focus on the behavior of cells as they organize to form chondrocranial cartilages and associated dermal bone primordia in the embryonic mouse head, advancing the coordination of chondrocranial cartilage and dermal bone to earlier phases of development. Our study advocates for a reassessment of the traditional definition of intramembranous ossification as a process that lacks any cartilage involvement and suggests a mechanistic basis for the observed tendency for chondrocranial cartilage and dermal bone to vary jointly in a coordinated manner.

## 2 MATERIALS AND METHODS

### 2.1 Mice

Mice were produced, sacrificed, and processed in compliance with animal welfare guidelines approved by the Pennsylvania State University Animal Care and Use Committee (#46558). Based upon timed mating and evidence of pregnancy, litters were sacrificed and collected as appropriate. Mice were housed in conventional cages (plastic rectangular tank; up to five adults) and placed in individually ventilated racks with corncob bedding, 12:12 h light:dark cycle, ad libitum food and water access, and environmental enrichment including nesting shredded paper and plastic toys. Bedding was changed once a week. Mice were assessed daily for illness or injury.

#### 2.1.1 Production of the R2Tom transgenic mouse line

A 557‐nucleotide sequence (*+210RUNX2* enhancer) in the last intron of the human *RUNX2* gene was shown to introduce gene expression in osteoblasts at early differentiation stages in zebrafish (Knopf et al., 2011; Weber, 2013). To label these osteoblasts in mice, we made a transgenic vector by joining this sequence, the mouse *Hsp68* minimal promoter sequence (Kothary et al., 1989), and the tdTomato gene sequence (Shaner et al., 2004). These three sequences were initially amplified by PCR: The *+210RUNX2* enhancer sequence (NC_000006.12: 45538559_45539115) was amplified from human DNA using 5’‐AATTTGGCTCCATGTTTTGG‐3′ and 5’‐GGCAGGCAGTAGATGTGTGA‐3′ primers; the *Hsp68* minimal promoter sequence was amplified from the Hsp68‐LacZ‐Gateway vector (Addgene plasmid #37843) (Pennacchio et al., 2006) using 5′‐ CCCGAATTCCGAGCTCCAGGAACATC‐3′ (hsp‐up) and 5’‐TTGCTCACCATGGCGCCGCGCTCT‐3′ (hsp‐dwn) primers; and the tdTomato gene sequence was amplified from the tdTomato‐N1 vector (Addgene plasmid #54642) using 5’‐CGGCGCCATGGTGAGCAAGGGCGA‐3′ (tom‐up) and 5’‐TTAGGATCCACGCCTTAAGATACATT‐3′ (tom‐dwn) primers. The amplified *+210RUNX2* enhancer sequence was cloned into the *Eco*RV site of the pBluescript II SK(+) plasmid vector (Stratagene). The amplified *Hsp68* minimal promoter and the tdTomato gene were mixed and used for the template of PCR using the hsp‐up and tom‐dwn primers. Because the other two PCR primers, hsp‐dwn and tom‐up, contain complementary sequences (underlined), some of these two PCR products anneal at the complementary region, and the entire complementary strands can be synthesized during PCR. As a result, the *Hsp68* minimal promoter and the tdTomato gene sequences join with the complementary region. This PCR product and pBluescript II SK(+) plasmid vector that contains the +*210RUNX2* enhancer sequence were both digested with *Eco*RI and *Bam*HI and ligated. The resultant construct, the *+210Runx2* enhancer, *Hsp68* minimal promoter, and tdTomato gene sequences, was confirmed by determining the nucleotide sequence. This plasmid was digested with *Hin*dIII and *Bam*HI and used for pronuclear injection to obtain transgenic mice in Mouse Genetics CoRE of the Icahn Medical Institute at Mt. Sinai. Positive transgenic mice were screened by PCR using 5’‐GACCAGCCTTCCCCAGAGCA‐3′ (hsp‐up2) and 5’‐CCGGGCAGTTGCACGGGCTTCT‐3′ (tom‐dwn11) primers.

To label osteoblasts and their precursors at varied differentiation stages, these mice (R2Tom) were crossed with Osx‐GFP mice (Rodda & McMahon, 2006) obtained from the Jackson Laboratory (strain #006361). In Osx‐GFP mice, cells expressing the *Osx* gene are labeled by the green fluorescent protein (GFP). Thus, in R2Tom;Osx‐Cre double transgenic mice, the *tdTomato* gene expression (red) is induced by the *+210Runx2* enhancer, while the *GFP* gene is expressed in cells that endogenously express the *Osx* gene.

#### 2.1.2 Mice used to determine cell derivation of chondrocranial cartilages

Wnt1‐Cre2 (Lewis et al., 2013) were purchased from the Jackson laboratory (strain #022137), and Mesp1‐Cre mice, established by Prof. Saga at the National Institute of Genetics (Saga et al., 1999), were obtained from Dr. Bruneau of the University of California‐San Francisco. To trace cells of neural crest origin and cells of mesodermal origin, R26R (Soriano, 1999) females, purchased from the Jackson laboratory (strain #003474), were bred with Wnt1‐Cre2 males and Mesp1‐Cre males, respectively. Embryos (E13.5–E15.5) obtained from dams were frozen in isopropanol chilled with liquid nitrogen, embedded in O.C.T. compound (Tissue‐Tek) on clashed dry ice, cryosectioned at 14 μm thickness (using Gold Microtome Blades, C. L. Sturkey Inc.), and mounted on glass slides (Superfrost Plus Microscope Slide, Fisher) (Bauer, 2010). These sections were then processed for lacZ staining (Bonnerot & Nicolas, 1993). To map the dynamic cranial neural crest/paraxial mesoderm boundary, we used four Mesp1‐Cre and three Wnt1‐Cre2 animals aged E13.5, three Mesp1‐Cre and one Wnt1‐Cre2 embryo aged E14.5, and two Mesp1‐Cre and two Wnt1‐Cre2 aged E15.5. These primary data were used in conjunction with published data (Kuroda et al., 2023; McBratney‐Owen et al., 2008).

#### 2.1.3 Histological analysis

Coronal sections of mouse embryos were made as we described previously (Pitirri et al., 2022). Some sections were stained with Alcian blue, picrosirius red, and hematoxylin based on the published protocol (Gruber et al., 2002), while other sections were used for immunohistochemical analysis to detect the GFP and tdTomato proteins. We used an anti‐GFP antibody (Abcam, ab183734; 1:400 dilution) and an anti‐tdTomato antibody (SICGEN, AB8181‐200, 1:400 dilution) as the primary antibodies, and the immunoreactions were amplified using the VECTASTAIN ABC HRP kit (Vector Laboratories, PK‐4001 and PK4005).

To determine the relative distribution of the GFP‐to‐tdTomato proteins, images of histological sections were opened as PDF files in Fiji (Schindelin et al., 2012). The scale was determined and set according to the scale included in the histological slice image. The color threshold was adjusted, and areas of interest were selected by an expert user. For each image slice, threshold color parameters were set to hue: 1–255; saturation 0–255; brightness 0–255 to mask and select regions of interest. The area was then measured using the measure function.

### 2.2 eMOSS: embryonic mouse ontogenetic staging system

Harvesting age measures the time elapsed between conception and collection of an embryo based on timed matings and is routinely used in experimental work because it is a simple metric that is easy to apply in practice. A single harvesting age is recorded for all embryos in a litter but differences in developmental progress exist among littermates (Miyake et al., 1996). The variation introduced by the use of harvesting age can affect our understanding of the timing and sequence of important developmental processes and events (e.g., initiation of cell differentiation, migration, or death; expression of a particular marker of a developmental process) creating an obscured confounding factor in understanding morphogenesis (Musy et al., 2018). Developmental staging expresses the maturity of an embryo through estimates of the amount of progress that an individual embryo has made along its ontogenetic trajectory based on select phenotypic characters. For specific mice, developmental ages were estimated using the embryonic Mouse Ontogenetic Staging System (eMOSS) (https://limbstaging.embl.es/) that compares the 2D outline of an embryo hindlimb with a library of mean hindlimb shapes for each hour of development between E9 and E15 to provide an estimate of an embryo's developmental age (Musy et al., 2018). Whenever possible, both the left and right hindlimbs were staged and their average was used to estimate developmental age. For any specimen older than E15 and for some younger specimens, it was not possible to estimate developmental age using this system. When developmental age is not available, we provide the standard embryo harvesting day for the specimen (e.g., E13.5). eMOSS age estimates (hereafter called developmental stages, or DS) are scaled to developmental timing, so that developmental ages are provided as point estimates in embryonic days and hours expressed as total hours (e.g., E12 = DS288), with an associated confidence interval of ±2 h. eMOSS estimates can be generated rapidly, since the method is scale‐invariant and only a picture of the hindlimb is required to establish a developmental stage estimate (Flaherty & Richtsmeier, 2018).

### 2.3 Computed tomography imaging

Micro‐computed tomography (microCT) images are primarily used in this study for visualization.

#### 2.3.1 Imaging and visualization protocols

MicroCT images for bone (E15.5 and older) and PTA‐enhanced microCT images for soft tissue visualization were acquired by the Center for Quantitative Imaging at the Pennsylvania State University (www.cqi.psu.edu) using the General Electric v|tom|x L300 nano/microCT system. Image data were reconstructed on a 2024 × 2024 pixel grid as a 32‐bit volume but may be reduced to 16‐bit volume for image analysis using Avizo 2021.2 (ThermoFisher Scientific, Waltham, MA). Scanning parameters varied from 60 to 100 kV and 75 to 170 μA to accommodate age group and type of scan performed. Voxel sizes ranged from 6.9 to 15 microns (μm) for bone scans and 4.5–8 μm for PTA‐enhanced scans. Tissue segmentation from microCT images was performed as previously described (Motch Perrine et al., 2022; Zheng et al., 2020). Three‐dimensional (3D) volume renderings of surface models of embryonic chondrocrania, Meckel's cartilage, and bone were visualized in Avizo 2021.2 and used for illustration here.

### 2.4 Large field microscopy

We used the Zeiss Axio Zoom V16 zoom microscope that combines a 16× zoom with a high numerical aperture of NA 0.25, achieving a very high aperture in any zoom range, allowing views of complete model organisms in fluorescence contrast, as well as observations down to the micron range. We used large‐field, high‐resolution fluorescent microscopy, ranging between 12.5× and 112×, to capture the expression of *RUNX2* (expression introduced by the *+210Runx2* enhancer; tdTomato) and *Osx* (GFP) in osteoblasts and their precursor cells in R2Tom;Osx‐GFP whole mouse embryos (see section 2.1 Mice, above). Large field microscopy work focused on cells of the forming frontal and parietal bones in R2Tom;Osx‐GFP whole mouse embryos.

## 3 RESULTS

### 3.1 CNC‐PM boundaries of the chondrocranium

Mesenchymal cells that contribute to bones of the lateral wall of the cranial vault in mice (frontal, parietal, squamosal) are derived from two distinct cell populations: the frontal and squamosal are derived from CNC (derived from the neuroectoderm) and the parietal is derived from PM (from the mesoderm germ layer) (Jiang et al., 2002; Yoshida et al., 2008). Differences in the osteogenic potential of CNC and PM cells have been proposed (e.g., Doro et al., 2019; Galea et al., 2021; Glaeser et al., 2021; Li et al., 2010; Senarath‐Yapa et al., 2013), but in vivo bones derived from CNC or PM are indistinguishable.

#### 3.1.1 Braincase floor

**FIGURE 3:**
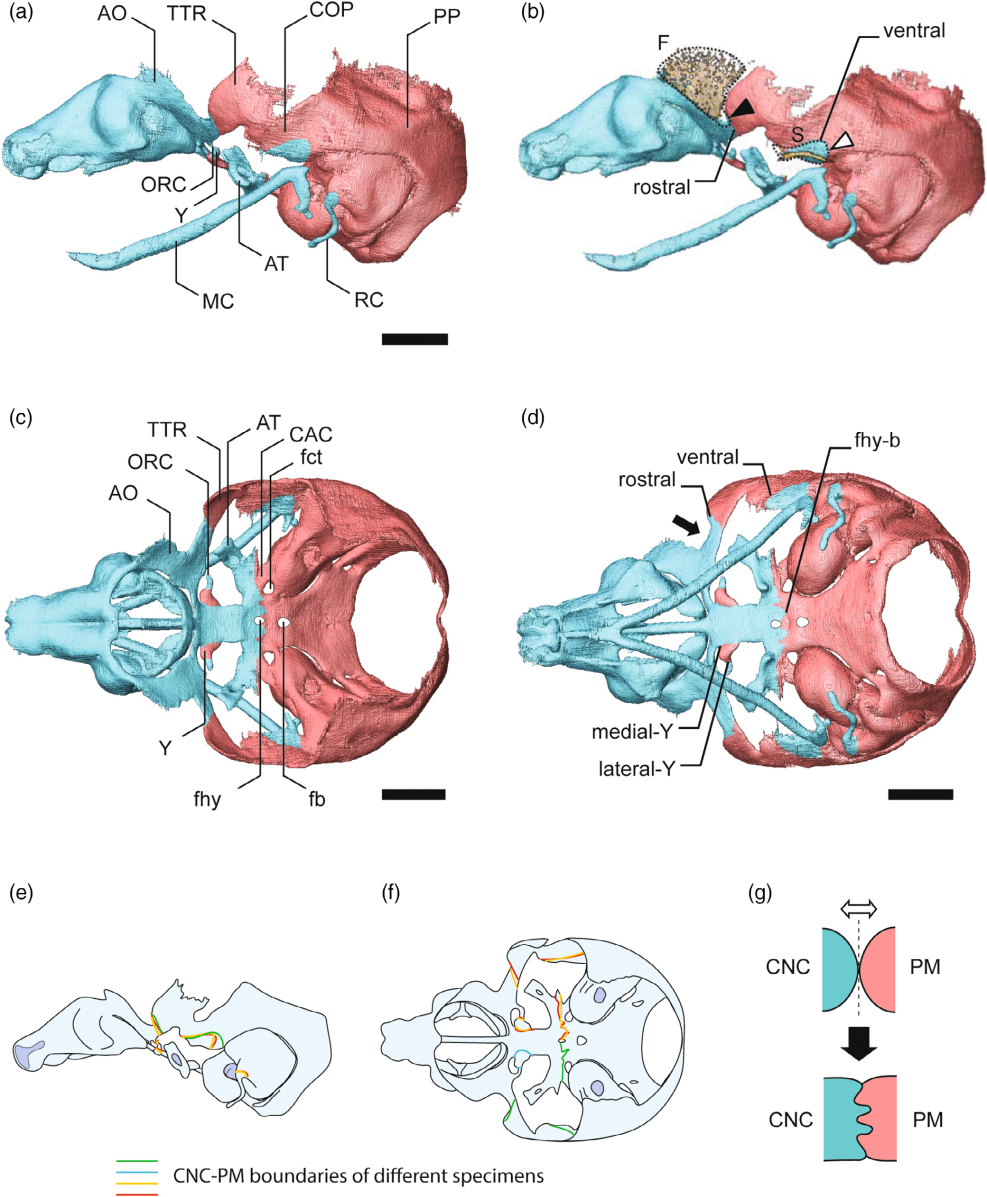
Distribution of the CNC‐derived (blue) and PM‐derived (pink) cells in the chondrocranium and part of the pharyngeal skeleton of the laboratory mouse at E15.5. A lateral view (a, with nose pointing to the left) shows the rostral and ventral CNC‐PM boundaries. Two other boundaries associated with the hypochiasmatic cartilage (Y) are found in the superior (c) and inferior views (d). We used Wnt1‐Cre2;R26R mice that mark CNC‐derived cells and Mesp1‐Cre;R26R mice that mark mesoderm‐derived cells to determine the derivation of particular cartilages. Results are visualized on the reconstruction of a chondrocranium (PTA‐enhanced microCT images) of a mouse embryo at E15.5. The basicranial fenestra (fb) and hypophyseal fenestra (fhy) were not detected in microCT images but added to the superior (c) and inferior (d) views at the positions observed by our histological analysis. (b) 3D reconstruction of the frontal (F) and squamosal (S) bones, obtained from microCT images of the same mouse shown in a,c,d. Only well‐mineralized regions are captured by microCT. Weakly mineralized regions, deduced from our histological analysis, are shown by dotted lines. Note that the rostral and ventral CNC‐PM boundaries partly overlap with the caudoventral edge of the frontal bone (closed arrowhead) and the dorsal rim of the squamosal bone (open arrowhead), respectively. The CNC‐PM boundaries are fuzzy with notable individual variation. Scale bar = 1.0 mm. 3D distribution of the cellular contributions also available as Supplemental video. AO, ala orbitalis; AT, ala temporalis; CAC, alicochlear commissure; COP, orbitoparietal commissure; fb, basicranial fenestra; fct, carotid foramen; fhy, hypophyseal fenestra; MC, Meckel's cartilage; ORC, orbital cartilage; PP, parietal plate; RC, Reichert's cartilage; TTR, tectum transversum; Y, hypochiasmatic cartilage. (e, f) Variations in the CNC‐PM boundaries among four specimens shown in the lateral (e) and superior views (f). The boundaries determined using Wnt1‐Cre2;R26R mice are shown in blue and green, while those using Mesp1‐Cre;R26R mice are shown in red and orange. Not all boundaries were determined for each of these four specimens. (g) Formation of the CNC‐PM boundary. The initial formation of the CNC‐PM boundary (dashed line) changes (open double arrow) depending on the movement of CNC‐ and PM‐derived cells, rate of their growth, and various other factors. These factors also affect the final CNC‐PM boundaries as seen in the undulating CNC‐PM boundary running through the hypophyseal fenestra (fhy‐b in d).

We used Wnt1‐Cre2;R26R and Mesp1‐Cre;R26R transgenic mice to determine the boundary between CNC‐derived and PM‐derived cells (CNC‐PM boundary) among cranial cartilages (Figure 3a–d) (embedded Video 1). Based on LacZ‐stained serial sections made from Wnt1‐Cre2;R26R and Mesp1‐Cre;R26R transgenic mouse specimens, we found that in principle CNC‐derived cells occupy the region rostral to the hypophyseal fenestra (fhy in Figure 3c) in the braincase floor (cranial base), while PM‐derived cells reside more posteriorly. Our identification of this CNC‐PM boundary of the braincase floor (fhy‐b in Figure 3d) is consistent with the results of previous studies, including the PM‐derived alicochlear commissure (Kuroda et al., 2023; McBratney‐Owen et al., 2008). However, we found marked, and we think significant, individual variability and highly complicated, undulating patterns of this boundary (Figure 3e, f). Variability of these wavy boundaries is likely due to individual differences in (1) timing, route, and duration of CNC and PM‐derived cell population movement: (2) rate of cell division and directions of growth; and (3) timing of onset or duration of the condensation stage affecting condensation size and emergent positioning of cells. Idiosyncratic differences in the timing of these biological processes explain individual variation in the position of the CNC‐PM boundaries among specimens (Figure 3g). The undulating CNC‐PM boundary (fhy‐b, Figure 3d) that intersects the hypophyseal fenestra (fhy, Figure 3c) can also be accounted for by differences in these processes. Once chondrocytes are surrounded by the rigid cartilage matrix, the CNC‐PM boundary becomes largely fixed regardless of the specific contour of a local frontier (Figure 3g).

**VIDEO 1:** Three‐dimensional reconstruction of the chondrocranium and part of the pharyngeal skeleton of the laboratory mouse at E15.5 showing the distribution of CNC‐derived (blue) and PM‐derived (pink) cartilages and CNC‐PM boundaries as determined using LacZ‐stained serial sections made from Wnt1‐Cre2;R26R and Mesp1‐Cre;R26R transgenic mouse specimens.

#### 3.1.2 Lateral wall

Our analyses confirmed four CNC‐PM boundaries in the lateral wall (rostral, ventral, medial‐Y, and lateral Y in Figure 3b, d), but we found these boundaries to be complicated, showing individual variation (Figure 3e, f). Among these four CNC‐PM boundaries, medial‐Y and lateral‐Y (Figure 3d), are associated with the PM‐derived hypochiasmatic cartilage (Y in Figure 3c; alae hypochiasmatica in Kuroda et al., 2023). These two CNC‐PM boundaries occupy the prechordal region, significantly rostral to the undulating CNC‐PM boundary that intersects the hypophyseal fenestra (fhy‐b in Figure 3d) of the braincase floor. We identify two additional CNC‐PM boundaries of the lateral wall, previously detected by (Kuroda et al., 2023), and refer to them as the rostral and ventral CNC‐PM boundaries (Figure 3b, d).

The rostral CNC‐PM boundary of the lateral wall lies near the union of the ala orbitalis with the tectum transversum (TTR in Figure 3b, d). Like the two CNC‐PM boundaries associated with the hypochiasmatic cartilage (Y in Figure 3a, c, d), the rostral boundary resides in a prechordal region significantly rostral to the CNC‐PM boundary of the braincase floor. Although our identification of these CNC‐PM boundaries is generally consistent with the findings of Kuroda and colleagues (Kuroda et al., 2023), we stress that the rostral boundary (Figure 3b, d) is convoluted and varies among specimens (Figure 3e, f). The anterior (apical) end of the rostral boundary of the lateral wall is located slightly dorsal to the narrowest region (black arrow in Figure 3d) of the union of the ala orbitalis (AO in Figure 3a, c) and the tectum transversum, and the posterior (basal) end is located caudal to the orbital cartilage (ORC in Figure 3a, c). Consequently, we conclude that the rostral CNC‐PM boundary runs within a ventral region of the tectum transversum, slightly dorsal to where it meets with the caudal end of the ala orbitalis (Figure 3b, d). The ventral CNC‐PM boundary lies on the ventral margin of the orbitoparietal commissure (Figure 3b, d) defining an island of CNC‐derived cartilage surrounded by PM‐derived cartilage composing a posterior portion of the lateral wall. Finally, a narrow caudal margin of the ala temporalis located rostral to the CNC‐PM boundary is often derived from PM, though this is an element of the pharyngeal skeleton rather than the chondrocranium (Figure 3f).

#### 3.1.3 Developmental implications of CNC‐PM boundaries

Our work confirms previous descriptions of salient irregularities in these boundaries (Kuroda et al., 2023; McBratney‐Owen et al., 2008) and adds new information (Figure 3e, f). Key to our argument of a link between the chondrocranium and dermatocranium, these irregularities contain information critical to the developmental basis of the temporospatial relationship between specific chondrocranial elements and the formation of dermal bones of the cranial vault (Table 1) (Kawasaki & Richtsmeier, 2017; Pitirri et al., 2020). At E15.5, prior to degradation of certain chondrocranial cartilages and as mineralization of the dermatocranium is initiated, the rostral CNC‐PM boundary spatially coincides with the caudoventral edge of the CNC‐derived frontal bone, and the inner surface of the caudoventral margin of the frontal bone overlies the CNC‐derived ventral region of the tectum transversum (Figure 3b, black arrowhead). This consistent temporospatial correspondence between cranial cartilage and dermal bone with common derivations, even in the presence of variability of the boundaries among specimens, suggests a developmental association between the rostral CNC‐PM boundary and the posterior limit of the CNC‐derived frontal bone. Similarly, the ventral CNC‐PM boundary spatially coincides with the dorsal rim of the CNC‐derived squamosal bone, and the dorsal region of the squamosal lies atop the CNC‐derived ventral margin of the orbitoparietal commissure (Figure 3b, open arrowhead). The rostral and ventral CNC‐PM boundaries flank the PM‐derived tectum transversum and orbitoparietal commissure, respectively, both of which are associated with the PM‐derived parietal bone. The parietal bone initially develops on the surface of the dorsal region of the tectum transversum (PM‐derived) and the anterior rims of this cartilage and bone pair align with each other at the frontier of the imminent coronal suture (Kawasaki & Richtsmeier, 2017).

**TABLE 1:** Dermal bones of the craniofacial skeleton, associated cranial cartilages and their derivation from CNC or PM. Individual cartilages of the mouse chondrocranium are identified in Figures 3.2 and 3.3 of Kawasaki and Richtsmeier (2017).

Cranial bones formed through intramembranous ossification and their derivation	Associated chondrocranial cartilages and their derivation
Frontal (CNC)	Ala orbitalis (CNC) and sphenethmoid commissure (CNC)
Parietal (PM)	Tectum transversum (CNC + PM), orbitoparietal commissure (CNC + PM), and parietal plate (PM)
Maxilla (CNC)	Pars intermedia (CNC), septum nasi (CNC), and paraseptal (CNC)
Lacrimal (CNC)	Pars intermedia (CNC) and paranasal process (CNC, not confirmed)
Premaxillae (CNC)	Pars anterior (CNC) and paraseptal (CNC)
Vomer (CNC)	Paraseptal (CNC), septum nasi (CNC), and lamina transversalis posterior (CNC)
Palatine (CNC)	Pila metoptica (CNC + PM), cupula nasi posterior (CNC), and presphenoid (CNC)
Pterygoid (CNC)	Hypophyseal (CNC + PM; E13.5‐E15.5) and alicochlear commissure (PM)
Interparietal (CNC + PM)	Parietal plate (PM) and tectum posterius (PM)
Squamosal (CNC)	Orbitoparietal commissure (CNC + PM) and tegmen tympani (PM)
Nasal (CNC)	Tectum nasi (CNC), pars anterior (CNC), cribriform plate (CNC, not yet formed prenatally)

Though CNC‐PM boundaries are more variable in the chondrocranium relative to the dermatocranium, with CNC‐PM boundaries running through some cartilages (Figure 3a–d), cells that contribute to members of the cartilage‐bone pairs of the lateral wall that we have identified are derived from the same embryonic source. These observations suggest that mesenchymal cells that migrate and condense in the same spatial domain give rise to chondrocytes and osteoblasts that form cartilage and dermal bone pairs and that their development, and the molecular signals that facilitate their development, may potentially be linked. To explore this possibility, we focus on how mesenchymal cells reach their target locations and differentiate in the lateral wall.

### 3.2 Condensation formation in the lateral wall and the impact of brain expansion

Migration of CNC‐derived mesenchymal cells occurs between E8.5 and E9.5 (Ferguson & Atit, 2019; Jiang et al., 2002; Yoshida et al., 2008). Jiang and colleagues (Jiang et al., 2002) showed that at E9.5, a frontonasal CNC‐derived cell population surrounds the frontonasal process and extends to the maxillary and mandibular CNC‐derived cell populations. Subsequently, mesenchymal cells in the subectodermal mesenchymal layer of the lateral wall of the brain condense to give rise to CNC‐derived frontal, PM‐derived parietal, and CNC‐derived squamosal domains by E10.5. As the brain continues to expand, the amplified growth of the frontal lobes transports these three domains rostrally, rearranging initial boundaries and creating dorsal extensions of the domains by E13.5 that extend toward the rostral hindbrain. Kuroda and colleagues further described that the frontier of PM‐derived cells in the lateral wall formed at E9.5 expands rostrally from the original position along the prechordal‐chordal border. This expansion explains the prechordal positions of the rostral CNC‐PM boundary and the two CNC‐PM boundaries associated with the hypochiasmatic cartilage (medial‐Y and lateral‐Y in Figure 3c, d) (Kuroda et al., 2023), as well as the individual variation in these boundaries (Figure 3e, f). Our observation that the rostral and ventral CNC‐PM boundaries of the lateral wall spatially coincide with the rims of overlying CNC‐derived dermal bones at E15.5 (Figure 3b) suggests that dermal bone‐chondrocranial cartilage associations (Table 1) are grounded in the mesenchymal cells from which they derive. These conclusions agree with Jiang and colleagues' (Jiang et al., 2002) assessment of a developmental series (E8.5–E17.5) that revealed the establishment of mesenchymal domains by E10.5 and subsequent rearrangement of CNC‐PM boundaries due to cerebral hemisphere expansion.

**FIGURE 4:**
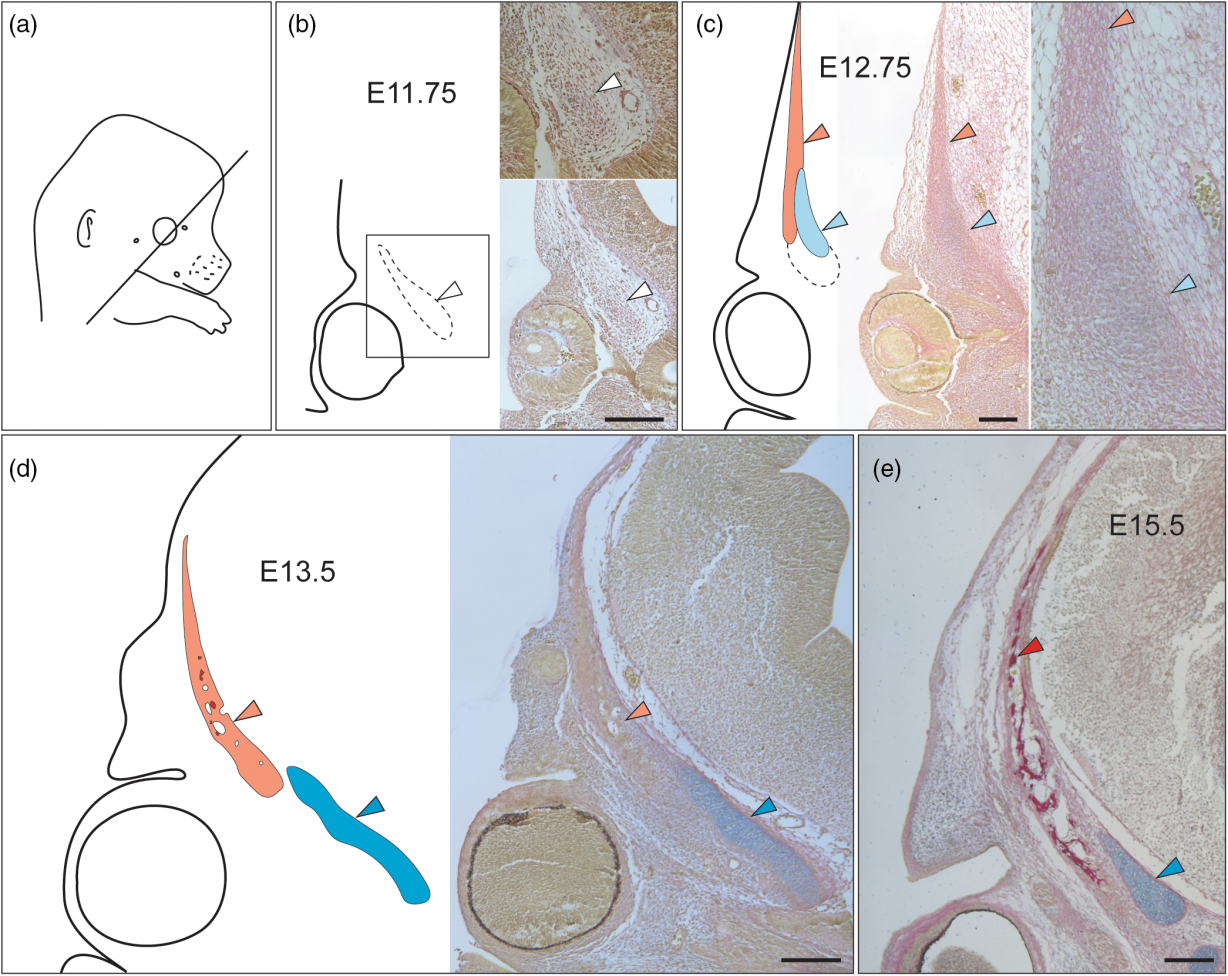
Developing frontal bone (pink or red) and ala orbitalis cartilage (light blue or blue) sectioned in a coronal plane (a) of embryos at E11.75 (b), E12.75 (c), E13.5 (d), and E15.5 (e). All sections were stained with Alcian blue, picrosirius red, and hematoxylin. A schematic drawing is shown on the right (b–d). The eye is illustrated as a circle in each schema, and arrowheads in each panel (b–d) indicate the same location of the undifferentiated (open), chondrogenic (light blue or blue), or osteogenic (pink) condensations. At E11.75 (b), a loose condensation of mesenchymal cells (dashed line), not stained in blue or red, is found above the eye. The squared region is enlarged at the upper right corner. At E12.75 (c), a condensed cell population above the eye is divided into two regions (subpopulations of cells), one weakly stained in blue (light blue) and the other weakly stained in red (pink). More loosely connected cells continuing basal to these two regions are shown with a dotted line. An enlarged view is shown on the right. At E13.5 (d), the chondrogenic condensation is clearly stained in blue. At this stage, the condensation stained weakly in red (pink) is spatially separated from the chondrogenic condensation (blue). This weak red condensation is well vascularized (open regions in the schema), and some strong red spots, presumably the initial bone matrix, are distributed frequently in the vicinity of blood vessels. At E15.5 (e), the periphery of the ala orbitalis cartilage is distinct (blue arrowhead) and surrounded by a fibrous tissue that is weakly stained in red, while the bone matrix is strongly stained in red (red arrowhead). Based on these observations, we interpret the two regions identified in the condensation at E12.75 (b) as follows: the region stained weakly in blue gives rise to the chondrogenic condensation, while the region stained weakly in red contributes to the osteogenic condensation. Note that these two regions are physically joined to each other at E12.75. By contrast, at E13.5 (d) and later (e), the bone and cartilage matrices are detectable as physically separate elements (see Figure 7). All scale bars measure 200 μm.

Separate streams of CNC‐derived cells migrate through the subectodermal space and give rise to the frontal domain and to the squamosal domain (Martik & Bronner, 2021). Similarly, a stream of PM cells migrates through the subectodermal space and gives rise to the parietal domain. After the migration, mesenchymal cells form a loose condensation in the frontal domain at E11.75 (Figure 4a, b), which subsequently splits into two separate condensations by E13.5 (Figure 4c–e). Whether cells differentiate into chondrocytes or osteoblasts depends upon their position, environmental cues, and their ability to respond to signals (Hall & Miyake, 1995). Based on (1) knowledge of the migration of CNC and PM cells and of the rearrangement of CNC‐PM boundaries as the cerebral hemispheres expand (Jiang et al., 2002; Yoshida et al., 2008); and (2) the temporospatial correlation between paired chondrocranial and dermatocranial elements that are either PM or CNC derived (Table 1), we propose that the rostral and ventral CNC‐PM boundaries of the lateral wall derive from borders established by E10.5 between the frontal, parietal, and squamosal mesenchymal domains.

### 3.3 Differentiation of chondrocytes and osteoblasts from CNC and PM mesenchyme

Our argument, however, is not consistent with the hypothesis that chondroprogenitors and osteoprogenitors differentiate before CNC migration (Akiyama et al., 2005). That hypothesis is based on the observation that the inactivation of *Osx* in *Sox9*‐expressing cells resulted in virtually no intramembranous bone formation. Since *Osx* and *Sox9* genes encode a transcription factor essential for osteogenesis and chondrogenesis, respectively (Komori, 2019; Lefebvre & Dvir‐Ginzberg, 2017; Sinha & Zhou, 2013), the lack of intramembranous bone when *Osx* is inactivated in *Sox9*‐expressing cells led to the hypothesis that all “osteo‐chondroprogenitor cells” are derived from *Sox9*‐expressing precursors (Akiyama et al., 2005). Mori‐Akiyama and colleagues (Mori‐Akiyama et al., 2003) found that transgenic mice in which *Sox9* is conditionally inactivated in CNC cells and their derivatives also failed to form CNC‐derived cartilage and endochondral bone, but all elements of the dermatocranium formed—though the elements were small and dysmorphic. The collective interpretation of these experiments was that if *Sox9* expression is essential for osteo‐chondroprogenitors, their segregation into osteoblast and chondroblast lineages must have occurred before the migration of CNC cells (Akiyama et al., 2005).

It is suggested that transcription factors SOX9 and MSX2 antagonistically regulate chondrogenesis (Semba et al., 2000; Takahashi et al., 2001). While SOX9 is indispensable for chondrogenesis, MSX2 represses the differentiation of *Sox9*‐expressing mesenchymal cells into chondrocyte precursors. As CNC‐derived cells migrate and differentiate into various cell types, the expression of *Sox9* becomes limited to chondrocyte precursors that form chondrogenic condensations (Akiyama et al., 2002; Eames et al., 2004; Takahashi et al., 2001). MSX2 represses differentiation of *Sox9*‐expressing mesenchymal cells into chondrogenic cells until epithelial‐mesenchymal interactions and/or other signals induce differentiation of specific *Sox9*‐expressing mesenchymal cells to chondrocyte precursors (Semba et al., 2000; Takahashi et al., 2001). Osteogenic condensations, on the other hand, are formed by *Runx2*‐expressing osteoprogenitors in which *Sox9* expression is downregulated (Day et al., 2005; Hill et al., 2005). Therefore, chondrogenic potency may be the default condition of mesenchymal cells (Goodnough et al., 2012; Hartmann, 2006; Shum & Nuckolls, 2001).

The experimental inactivation of *Sox9* in CNC cells (Mori‐Akiyama et al., 2003) apparently prevented the differentiation of CNC‐derived mesenchymal cells into chondrocytes but had little or no effect on osteoblast differentiation. Importantly, *Sox9* is also inactive in CNC cells and their derivatives in normal mice until the antagonistic function of MSX2 is downregulated (Day et al., 2005; Hill et al., 2005). It is thus plausible that the formation of CNC‐derived dermal bone in these transgenic mice is simply due to the upregulation of *Runx2* expression regardless of the expression of *Sox9* in CNC cells and their derivatives. If true, this experiment (Mori‐Akiyama et al., 2003) suggests that *Sox9* expression is not required for either the formation of osteogenic condensations or subsequent osteogenesis. The observation that the inactivation of *Osx* in CNC and PM cells and their derivatives expressing *Sox9* resulted in virtually no intramembranous bone (Akiyama et al., 2005), is most likely due to the ubiquitous expression of *Sox9* in CNC and PM cells of early mouse embryos (Lee & Saint‐Jeannet, 2011; Ng et al., 1997; Zhao et al., 1997). *Sox9*‐expressing CNC and PM cells may be precursors of various types of cells, not limited to chondrocytes or osteoblasts.

These considerations prompted us to adopt the hypothesis that in CNC‐derived mesenchyme chondrogenic condensations develop from mesenchymal cells that maintain *Sox9* expression, while osteogenic condensations arise from cells that upregulate *Runx2* expression. The experiment of Mori‐Akiyama et al. (Mori‐Akiyama et al., 2003) also demonstrates that chondrocranial cartilage is not necessary for dermal bone *formation*, but chondrocranial cartilage appears to be essential to the appropriate shaping of dermal bone.

### 3.4 Further differentiation of osteoblasts

During the formation of chondrocranial cartilage‐dermatocranial bone pairs, the osteogenic condensation separates from the chondrogenic condensation when recently differentiated osteoblasts of the osteogenic condensation begin to secrete bone matrix (Figure 4d). Both *Runx2* and *Osx* encode transcription factors critical to osteogenesis and their expression patterns are dynamic, changing in function and intensity during the various differentiation stages of osteoblasts and their precursors (Komori, 2019, 2020; Sinha & Zhou, 2013; Tabler et al., 2016). *Osx* also directly upregulates the expression of various bone matrix protein genes (Liu et al., 2020). *Runx2* enhances the proliferation of multipotent mesenchymal cells, osteoprogenitors, and preosteoblasts, augments the differentiation of mesenchymal cells to osteoblast precursors and subsequently to osteoblasts, and enhances the expression of bone matrix protein genes (Kawane et al., 2018; Komori, 2019, 2020). The expression level of *Runx2* is weak in uncommitted mesenchymal cells, upregulated in preosteoblasts, relatively increased and high in immature osteoblasts, and downregulated in mature osteoblasts (Komori, 2019). The expression of *Osx* is induced by *Runx2* in preosteoblasts, and *Osx* acts downstream of *Runx2* (Nakashima et al., 2002). The expression of *Osx* and *Runx2* is subsequently mutually regulated (Kawane et al., 2014) as both transcription factors enhance the differentiation of preosteoblasts into immature osteoblasts and regulate their maturation (Komori, 2019, 2020).

**FIGURE 5:**
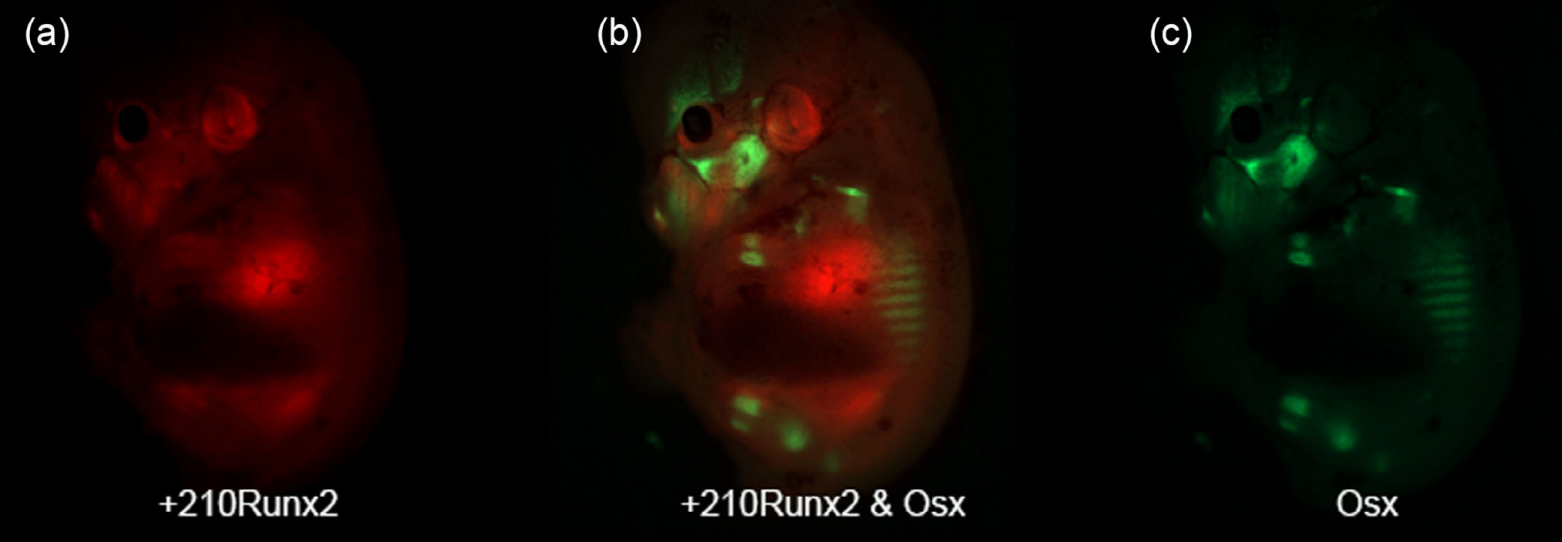
The R2Tom;Osx‐GFP mouse at E13.5 in which the expression of RUNX2 and Osx are detected with different fluorescent reporters in whole embryos visualized by large field microscopy, 12.5×. tdTomato (red) is used to detect RUNX2 gene expression and GFP (green) is used to detect Osx gene expression. a. tdTomato (red) expression; c. GFP (green) expression; and b. expression of both tdTomato (red) and GFP (green) fluorescent markers. GFP reveals the location of many emerging cranial and post‐cranial bones. The distribution of tdTomato largely overlaps with GFP where the bone is present, but because tdTomato expression is not limited to cells that contribute to bone, it is seen in the dermis of the ear, in hair follicles of the nose and above the eye, and in the heart.

In zebrafish, the *+210RUNX2* enhancer was shown to drive gene expression in osteoprogenitors surrounding the edge of the developing opercle bone (Weber, 2013). To determine whether this enhancer also drives gene expression in bone cells in mice, we made R2Tom transgenic mice, in which the *+210RUNX2* enhancer drives tdTomato gene expression. We then bred these mice with Osx‐GFP mice. In the resulting double transgenic mouse embryos (R2Tom;Osx‐GFP), fluorescent signals of both tdTomato (potentially labeling *Runx2*‐expressing cells) and GFP (labeling *Osx*‐expressing cells) were detected with different fluorescent reporters in whole R2Tom;Osx‐GFP embryos (Figure 5).

**FIGURE 6:**
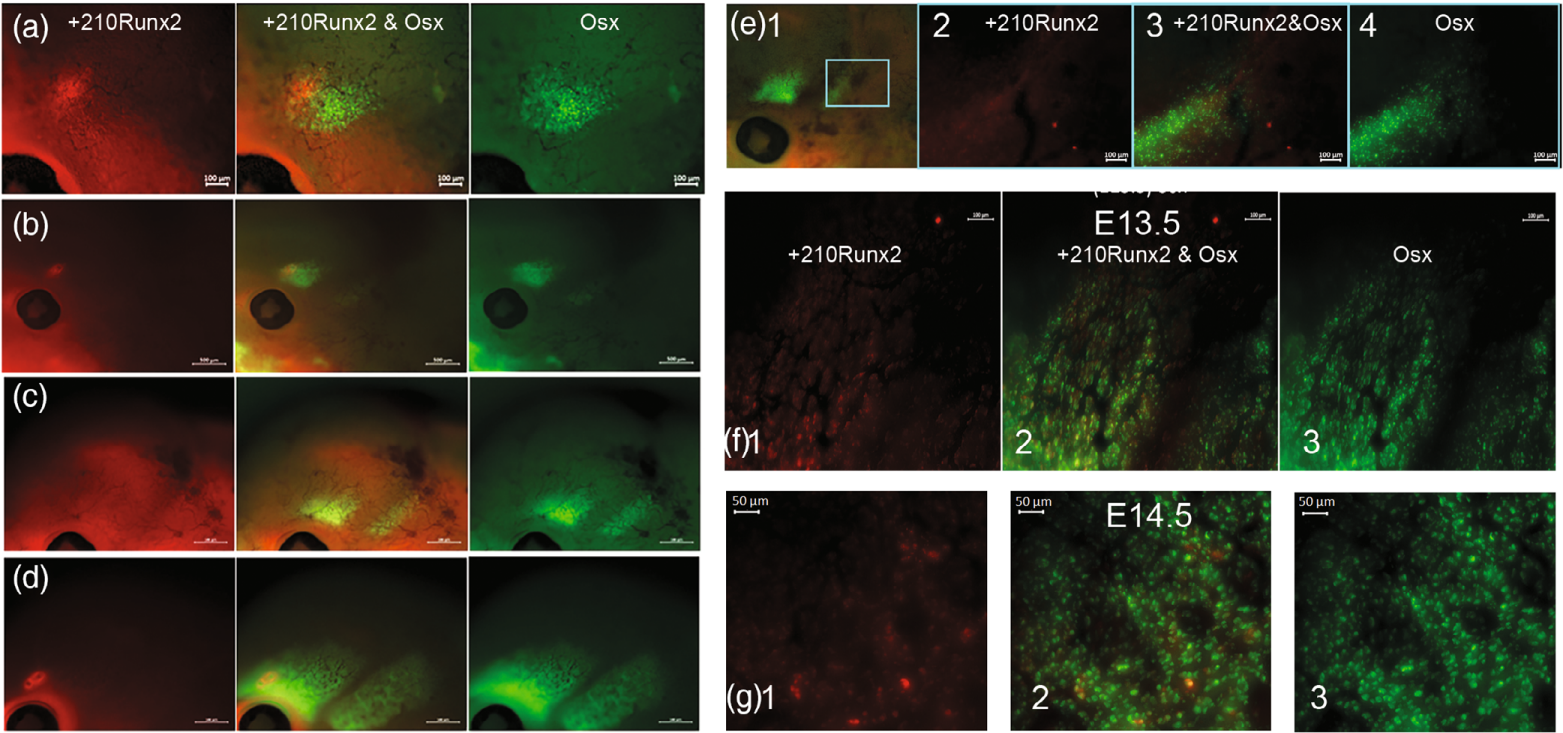
Developmental sequence of the coalescence of cells destined to differentiate into osteoblasts that form the frontal and parietal bones and the incipient coronal suture as visualized by large field microscopy in R2Tom;OsxGFP mice in which expression driven by the *+210RUNX2* enhancer and Osx are detected with tdTomato (red) and GFP (green) fluorescent reporters, respectively. Views show the organization of cells over developmental time prior to mineralization. (a–d) Embryonic age and developmental stage (DS) given in hours since conception shows rapidity of changes in the cellular organization of frontal and parietal bones and coronal suture. In each triplet (a–d), rostrum is to the left and eye can be seen in the lower left corner of each image, 25×. In each row, tdTomato (red) expression directed by +210Runx2 is shown in the left panel, GFP (green) expression directed by Osx is shown in the right panel, and the two fluorescent markers are shown in the center panel. a. embryo harvested at E12.5 and aged by our developmental staging system to be 287 h postconception (DS287) reveals the initial sign of cells expressing GFP organized as a dispersion of Osx + cells above the eye, 80×. The focal point of tdTomato fluorescence seen above the eye represents a hair follicle, confirmed by immunohistochemistry and not related to the cellular assembly of the frontal, so at this stage, cells forming the frontal are exclusively expressing GFP. (b) embryo harvested at E12.5 and staged to be DS 292.5 reveals cells expressing GFP forming the incipient frontal and in the preliminary stages of organization of the parietal (faint GFP expression). Lack of expression between these two cell clusters marks the future site of the coronal suture, 25×; (c) population of cells expressing tdTomato and Osx associated with the cellular assembly of the frontal and parietal at E13.5 and staged to DS 317. Frontal and parietal appear as two diffuse clouds of expression that expand apically forming a curved surface with a distinct division that marks the incipient coronal suture, 25×; (d) embryo harvested at E13.5 and staged to DS322 showing further organization and expansion of cells forming the frontal and parietal with cell organization beginning to show a honeycombed pattern. Absence of cells producing tdTomato and/or GFP indicates the location of the incipient coronal suture. Scale bar in a measures 100 μm; scale bars in b–d measure 500 μm. (e) Presumptive parietal region in a R2Tom;Osx‐GFP embryo at E12.5 determined by our developmental staging system to be DS307.5. e1—embryo head with cells already organized into frontal formation above the eye. The blue box indicates the area of incipient parietal bone formation shown at 80× in panels e2—tdTomato expression; e3—a dual expression of tdTomato and Osx; and e4—Osx expression. Note that the distribution of tdTomato largely overlaps with the distribution of GFP for cells that are organized to form the parietal bone. (f) Cells organizing to form the frontal and parietal bones of an R2Tom;Osx‐GFP embryo at E13.5 reveal that the distribution of tdTomato largely overlaps with GFP and that expressing cells largely avoid the area of the presumptive coronal suture. Scale bars measure 100 μm, rostrum to left, 80×. f1—tdTomato expression of incipient frontal and parietal bones with lack of expressing cells revealing coronal suture; f2—a dual expression of tdTomato and Osx; f3—Osx expression. (g) Cells organizing to form the presumptive parietal bone in a R2Tom;Osx‐GFP embryo at E14.5 (DS354.5) reveal a honeycombed or lattice‐like pattern with cells expressing tdTomato and GFP completely overlapping and organizing around voids presumably containing vessels and/or nerves. Visualized using large field microscopy, 80×, rostrum to the left, scale bars measure 50 μm. g1—tdTomato expression; g2—dual expression of tdTomato and Osx; g3—Osx expression.

We collected data from 66 R2Tom;Osx‐GFP specimens across three chronological ages: E12.5, E13.5, and E14.5. Nearly all mice were staged by our developmental staging system (Musy et al., 2018) revealing a range in the development stage (DS) across our sample from 262 to 370 h from conception (DS262–DS370). A subset of specimens ordered by DS provides the basic pattern of preosteoblast cells organizing and assembling the incipient frontal and parietal bones and the coronal suture (Figure 6a–d). R2Tom;OsxGFP mice reveal tdTomato and GFP signals in the area of the incipient frontal bone hours before signals are visible around the forming parietal (Figure 6a). Hours later (DS292 in 6b; DS307.5 in 6e), tdTomato and GFP signals are also clearly marking the cellular condensation of the formative parietal bone, and an incipient coronal suture is obvious. The distribution of tdTomato largely overlaps the distribution of GFP indicating that tdTomato is produced in preosteoblasts expressing *Osx* prior to matrix secretion by these cells (no bone matrix is detectable at E12.75; see Figure 4c). Approximately 1 day later at E13.5 (Figure 6f) preosteoblast cells of the emergent frontal and the emergent parietal bones are organized in a pattern that resembles a lattice, with cells arranged around open spaces, presumably around forming vasculature (Percival & Richtsmeier, 2013), and the incipient coronal suture is well defined. By E14.5 (DS354.5, Figure 6g), the distribution of tdTomato completely coincides with the expression of GFP within the lattice‐like arrangement of cells forming the parietal, and the number of the GFP signals is apparently larger than that of the tdTomato signals.

**FIGURE 7:**
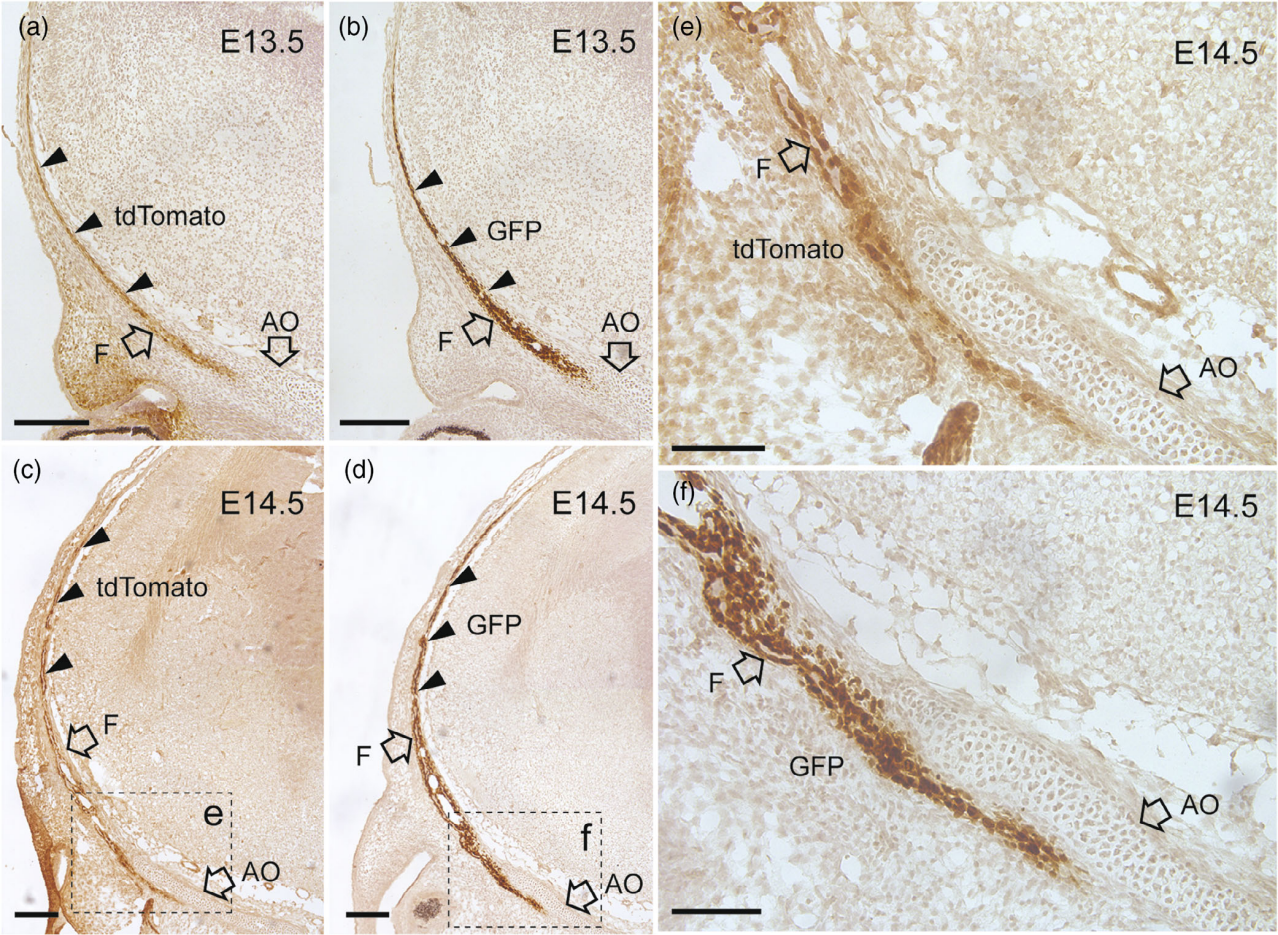
Immunohistochemical detection of the GFP and tdTomato protein in R2Tom;Osx‐GFP double transgenic mice. Coronal sections of E13.5 embryos (a, b) and E14.5 embryos (c, d) were analyzed using the anti‐tdTomato antibody (a, c) and anti‐GFP antibody (b, d). Each panel shows the frontal bone (F) and the ala orbitalis cartilage (AO) above the eye which is shown in the lower left corner (see Figure 4). The dashed rectangle region in c and d is enlarged in e and f, respectively. Immunosignals were detected in osteoblasts and their precursors as brown signals in the developing frontal bone. The bone matrix can be detected as a narrow region between two osteoblast layers (see Figure 4). Scales: 200 μm (a–d), 100 μm (e and f).

These mice were then sectioned in the coronal plane and investigated in more detail by immunohistochemical analysis. At E13.5, when the incipient frontal bone matrix was identified (Figure 7a, b), immunoreactivity for both tdTomato and GFP was detected on the outer and inner surfaces of the matrix, and the signal extended basally in the region immediately superficial to the apical edge of the ala orbitalis (AO in Figure 7), and apically in a thin layer between the epidermis and meninges (Figure 7a, b). Notably, no bone matrix was found at either the basal or apical ends of the matrix. At E14.5, while the bone matrix was noticeably expanded, similar immunoreactivity for tdTomato and GFP was detected on the superficial surface of the apical edge of the ala orbitalis stretching apically to a region without bone matrix (Figure 7c, d). Because GFP in this double transgenic mouse, as in Osx‐GFP mice, labels *Osx*‐expressing cells including osteoblasts and their precursors (Rodda & McMahon, 2006), these results show that the *+210RUNX2* enhancer drives gene expression in osteoblasts and their precursors in mice.

A strict differential distribution of immunoreactivity for tdTomato and GFP was not discernable along the apical‐basal axis of the frontal bone, as reactivity for both fluorescent reporters appeared in approximately the same locations. However, immunoreactivity for tdTomato was detected in a smaller number of cells relative to GFP. At E14.5, tdTomato was detected in almost all cells that are directly associated with the inner and outer surfaces of the bone matrix but tdTomato expression was limited in cells located distant from the matrix surface. GFP was also positive in cells of the matrix surface and within two or three additional layers of cells located deep into the matrix surface (Figure 7e, f). As a result, our study of the formative frontal bone indicated that the area occupied by tdTomato‐positive cells is ~71% of the area occupied by GFP‐positive cells (4.12 × 10^−2^ mm^2^ for tdTomato and 5.80 × 10^−2^ mm^2^ for GFP in Figure 7c, d). This observation suggests that a considerable proportion of tdTomato is made by osteoblasts that are located on the surface of the nascent bone matrix, presumably secreting the bulk of the bone matrix. On the other hand, cells producing GFP but not producing td‐Tomato are found two or three layers deep to the bone matrix surface and most likely represent osteoblast precursors that do not actively secrete the bone matrix. If this is true, counter to the consensus view of *RUNX2* expression (Long, 2012; Nakashima et al., 2002), it is likely that the *+210RUNX2* enhancer drives gene expression in osteoblasts and their precursors *after* the onset of *Osx* expression.

### 3.5 Integration of cranial cartilage and intramembranous bone

We previously proposed a functional relationship between chondrocranial cartilages and dermal bone based on observed spatiotemporal correspondences in their mutual development and statistical evidence of morphological integration of these tissues (Kawasaki & Richtsmeier, 2017; Motch Perrine et al., 2022; Pitirri et al., 2020). In the subectodermal space of the lateral wall of mouse embryos, multipotent mesenchymal cells migrate, undergo interactions with epithelial cells, and give rise to chondrogenic condensations closest to the meningeal layer of the brain and to osteogenic condensations in a more superficial layer closer to the epidermis. When separate condensations form from chondrogenic and osteogenic cells (Figure 4b, c) and overt cell differentiation begins (Figure 4d), an interface maintains separation of chondrocranial cartilages and dermatocranial bones, and these two different types of cells do not mix (Figure 4d, e). Chondroblasts differentiate first while cells committed to the osteoblast lineage proliferate and subsequently begin to secrete osteoid between two osteoblast layers. These dermal bone primordia are located on the edges of the superficial (outer) surface of already formed chondrocranial cartilages (Figure 4d, e). If, as we hypothesize, paired chondrocranial cartilages and dermal bones (Table 1) are formed by mesenchymal cells of the same domain, a developmental basis for the linking of specific cranial cartilage and dermal bone pairs is established during the migration of mesenchymal cells and subsequent formation of distinct domains (e.g., frontal, parietal, and squamosal), prior to the formation of chondrogenic and osteogenic condensations, pushing their connection to an earlier stage of skeletogenesis.

## 4 DISCUSSION

We have largely confirmed the CNC‐PM boundaries of chondrocranial cartilages in mice identified by other researchers (Kuroda et al., 2023; McBratney‐Owen et al., 2008), but stress that CNC‐PM boundaries within specific cranial cartilages can be unclear with notable individual variation at precise time points. A previous study (Jiang et al., 2002) revealed that populations of CNC‐derived and PM‐derived chondrocyte precursors are adjacent to one another early in development, but shift positions as the brain expands. If CNC‐ and PM‐derived cell populations shift their positions at different times, or if the onset or duration of the condensation stage differs between cell populations during this shift in positioning, differentiation of CNC‐ and PM‐derived chondrogenic cells could initiate at different times or occur at different rates thus affecting condensation size and emergent positioning of cells. Shifting of local boundaries according to idiosyncratic differences in the timing of these biological processes explains individual variation in the position of the CNC‐PM boundaries among specimens (Figure 3g). The undulating CNC‐PM boundary running through the hypophyseal fenestra (Figure 3c, d) can be accounted for by differences in growth rate or in local directions of growth of adjacent CNC‐ or PM‐derived chondrogenic condensations (Figure 3g). Once chondrocytes are surrounded by the rigid cartilage matrix, the CNC‐PM boundary becomes largely fixed regardless of the specific contour of a local frontier.

Our mapping of CNC‐PM boundaries of the chondrocranium contrasts with elements of the mouse skull where CNC‐PM boundaries commonly divide along osseous element margins (Jiang et al., 2002) that are determined relatively late in development when mineralization fronts are separated by either sutures or synchondroses. A notable exception found in mice is the interparietal bone composed of a CNC‐derived central portion and PM‐derived lateral portions (Jiang et al., 2002). The dual origin of the interparietal is thought to result from the fusion of the CNC‐derived postparietal bones and PM‐derived tabular bones during evolution (Koyabu et al., 2012). Because no separate bone plates composing the interparietal have been reported in laboratory mice, their CNC‐ and PM‐derived osteogenic populations likely coalesce before, or at the onset of matrix mineralization. Interparietal bone formation appears to be an example of the persistent loss of skull bones demonstrated over roughly 150 million years of synapsid evolution (Sidor, 2001). The mechanistic basis for why boundaries break down and sutures fail to form at the frontiers of bones that evolved as separate elements could provide insight into the modern disease of craniosynostosis (Flaherty et al., 2016; Richtsmeier et al., 2006).

Although tdTomato signals are generally weak relative to GFP and not always clearly detectable in double‐positive cells by large field microscopy, immunohistochemical analysis reveals that most cells lying on the surface of forming dermal bone matrix are positive for tdTomato (*+210Runx2*) and GFP (*Osx*) (Figure 7). Analysis of R2Tom;Osx‐GFP mice suggested that the *+210RUNX2* enhancer drives gene expression in osteoblasts and their precursors *after* the onset of *Osx* expression, a finding that is inconsistent with the consensus view of *RUNX2* expression (Long, 2012; Nakashima et al., 2002). Our findings also vary with the result of two studies that compared the *+210RUNX2* enhancer and an *osterix* enhancer in zebrafish (Knopf et al., 2011; Weber, 2013). During opercle bone formation, the *+210RUNX2* enhancer drives gene expression in surrounding cells, whereas the *osterix* enhancer drives gene expression in inner cells (Weber, 2013). Furthermore, after fin amputation, the *+210RUNX2* enhancer drives gene expression earlier than the *osterix* enhancer (Knopf et al., 2011). It is possible that the *+210RUNX2* enhancer drives gene expression in osteoblast precursors at slightly different differentiation timings in mice and zebrafish.

Our hypothesis of a temporospatial relationship between unmineralized chondrocranial elements and dermatocranial elements may be extrapolated to the ocular skeleton of reptiles and birds (sauropsids) and explain spatial relationships of scleral cartilage and ossicles. In various sauropsids, scleral ossicles and scleral cartilage form in close association with one another (Franz‐Odendaal, 2020) but scleral ossicles ossify intramembranously, independent of scleral cartilage. The close relationship between scleral cartilage and ossicles in sauropsids may be explained if these cartilage and ossicles develop from different condensations that originated from the same domain of mesenchymal cells, as we hypothesize for specific pairs of chondrocranial and dermatocranial elements. In contrast, scleral ossicles of teleost fishes form through perichondral ossification (Franz‐Odendaal et al., 2007) and the homology of scleral ossicles in teleosts and those in sauropsids is controversial (Franz‐Odendaal & Hall, 2006). If our speculation is correct, osteogenic condensations that give rise to sclerotic ossicles were secondarily lost in the teleost lineage (Franz‐Odendaal & Hall, 2006) while the chondrogenic condensation evolved to give rise to perichondral ossicles. These arguments support the hypothesis that sauropsid sclerotic ossicles are not homologous to teleost sclerotic ossicles.

Previous work from our group and the data presented here provide a foundation for further testing of the hypothesis of a mechanistic relationship between chondrocranial cartilage and dermal bone development. Our findings of the dynamic nature of the CNC‐PM boundary in mice infer that mesenchymal cells that migrate and condense to define the frontal, parietal, and squamosal domains give rise to cartilage and dermal bone pairs and that the signals that facilitate their organization may be integrated. Our work has helped to clarify the roles of *Sox9* and *Runx2* in the establishment of chondrogenic and osteogenic condensations and generated the hypothesis that chondrogenic condensations develop from CNC‐derived mesenchymal cells that maintain *Sox9* expression, while mesenchymal cells that upregulate *Runx2* expression are involved in osteogenic condensations. The experiments of Mori‐Akiyama and colleagues (Mori‐Akiyama et al., 2003) suggest that osteogenic condensations form without chondrogenic condensations. Though the condensation of chondrocytes and subsequent cartilage formation is not involved in the organization of osteogenic condensations or dermal bone formation, cartilage‐dermal bone pairs are established prior to overt differentiation and when cartilage is not present, dermal bones are small and malformed. Combining our findings with the fact that the primary function of skeletal connective tissue is to provide support and protection, in this case for the brain and other cranial sense organs, we propose that chondrocranial cartilages contribute to the appropriate mechanical strength of the lateral wall during early developmental stages and also during late developmental stages by guiding the proper shaping of the dermatocranium. Morphogenesis, the process by which bones of the skull take shape, occurs after the formation of osteogenic condensations. Chondrogenesis appears to contribute to the proper morphogenesis of dermal bones.

## 5 CONCLUSIONS

Our original work emphasized a temporospatial association between chondrocranial elements that do not mineralize endochondrally or perichondrally and dermal bones that form through intramembranous ossification (Kawasaki & Richtsmeier, 2017; Pitirri et al., 2020). Here we propose that a relationship between chondrocranial cartilage and dermal bone begins early in development during the migration of mesenchymal cells and progresses through the subsequent formation of mesenchymal domains—prior to the differentiation of chondrocytes and osteoblasts. Mesenchymal cells that migrate together and then give rise to the frontal, parietal, and squamosal domains contain both chondrogenic and osteogenic precursors. Once mesenchymal domains are formed, a deep subpopulation of cells located close to the meningeal layer of the brain forms a chondrogenic condensation, while a more superficial subpopulation of cells, closer to the epidermis, forms an osteogenic condensation. As the brain expands and the matrix of cartilage and bone is secreted, the edges of osteogenic condensations sit on the ectocranial surface of the more internally situated cartilages (Figures 4 and 7), which may function as scaffolds for the initial development and shaping of dermal bones in the mouse head. This integration of chondrocranial cartilage and dermal bone may also function to ensure mechanical strength of the lateral wall as cartilages dissolve and bone begins to mineralize during the significant and rapid expansion of the frontal lobes and suggests a mechanistic basis for the supervision of the proper shaping of dermal bones (Kawasaki & Richtsmeier, 2017; Motch Perrine et al., 2022; Pitirri et al., 2020).

## AUTHOR CONTRIBUTIONS

**M. Kathleen Pitirri:** Investigation; methodology; validation; visualization; data curation. **Joan T. Richtsmeier:** Conceptualization; investigation; funding acquisition; writing – original draft; methodology; validation; visualization; writing – review and editing; project administration; data curation; supervision. **Mizuho Kawasaki:** Visualization; validation; formal analysis; data curation; writing – review and editing. **Abigail P. Coupe:** Data curation; formal analysis; investigation; writing – review and editing; validation; visualization. **Susan Motch Perrine:** Visualization; writing – review and editing; software; data curation; investigation; writing – original draft. **Kazuhiko Kawasaki:** Conceptualization; investigation; writing – original draft; methodology; validation; visualization; writing – review and editing; formal analysis; data curation; supervision.

## FUNDING INFORMATION

This research was supported, in part, by NIDCR/NIH R01 DE027677, R01DE 029832, R01DE031439, and NICHD/NIH P01 HD078233.

## CONFLICT OF INTEREST STATEMENT

The authors declare no conflict of interest.

## Data Availability

MicroCT data used in visualizations are available through the Penn State University Libraries ScholarSphere repository at: https://doi.org/10.26207/qgke-r185 and include bone microCT images, PTA‐enhanced microCT images, and 3D reconstruction examples of chondrocrania. PTA‐enhanced staining protocols for various embryonic ages of mice are available at https://doi.org/10.1002/dvdy.136. Code for automatic chondrocranium segmentation with very sparse annotation via uncertainty‐guided self‐training is available through https://github.com/ndcse-medical/CartSeg_UGST. The R2Tom mouse line is available as sperm from JAX. All other data are available upon request.
